# Improving outcomes in total knee arthroplasty—do navigation or customized implants have a role?

**DOI:** 10.1186/s13018-016-0396-8

**Published:** 2016-05-23

**Authors:** Matthew D. Beal, Dimitri Delagramaticas, David Fitz

**Affiliations:** Northwestern University Feinberg School of Medicine, Chicago, USA

## Abstract

Modern total knee arthroplasty is effective at treating the pain and disability associated with osteoarthritis. The number of total knee replacements done in the USA continues to increase. Despite the great care taken during all of these procedures, some patients remain dissatisfied with their outcome. While this dissatisfaction is likely multifactorial, malalignment of the prosthetic components is a major cause of postoperative complications. A neutral mechanical axis plus or minus 3° is felt to have a positive impact on the survivorship of the prosthesis. Conventional instrumentation has been shown to have a significant number of total knee replacements that lie well outside a neutral coronal alignment. With that in mind, significant effort has been placed into the development of technology to improve the overall alignment of the prosthesis. In order to reduce the number of outliers, several companies have developed cost-effective systems to aid the surgeon in achieving a more predictably aligned prosthesis in all three planes. We will review the literature that is available regarding several of these tools to examine if navigation or custom guides improve outcomes in total knee arthroplasty. Our review supports that while both navigation and custom implants guides seem to be a cost effective way to achieve a predictable mechanical alignment of a total knee prosthesis therefore reducing the number of outliers, the cost may be increased operative times with no perceived difference in patient satisfaction with navigation custom guides.

## Background

Osteoarthritis of the knee is among the most common contributing causes of global disability, prevalent in as much as 20 to 40 % of those over 75 years of age [1, 2]. Total knee arthroplasty (TKA) has revolutionized the quality of life for millions of patients and has proven to be a cost-effective and reliable treatment for symptomatic osteoarthritis of the knee. An estimated 700,000 TKAs are performed in the USA each year, making it one of the most common procedures annually performed, with a projected increase in demand to over 3.48 million procedures by 2030 [3–5].

Despite the overwhelming successes of TKA, several studies have found that about 1 in 5 patients undergoing TKA are dissatisfied with the results of their surgery [6]. Various aspects of TKA have been implicated to explain this phenomenon, one of which being implant positioning, where deviations greater than 3° from goal alignment have been correlated with worse clinical outcomes, abnormal wear, premature loosening, and early implant failure [7, 8]. As such, achieving as close to neutral mechanical alignment in the coronal plane remains the gold standard goal for implant positioning. A method for consistently achieving this goal, however, remains somewhat elusive. Mason et al., for example, demonstrated that conventional instrumentation fails to achieve within ±3° of the mechanical axis greater than 30 % of the time [9]. Drawbacks to conventional alignment systems include difficulty with identifying anatomic landmarks intraoperatively as well as the assumption of standard anatomic relationships, which may not always be consistent across all patients.

Aiming to achieve a more precise and repeatable method of achieving the goals of painless and durable TKA, significant evolution in component design, material science, and technique of instrumentation has transpired since the first knee replacement was first performed in the late 1960s. Recent developments in computer-aided surgery and patient specific systems have contributed to the evolving aspect of instrumentation and implantation techniques. Robotic and handheld computer navigation systems, computer-guided cutting instruments, tibial trial sensors, and patient-specific cutting blocks have all seen significant recent development and attention within the TKA marketplace. Trends in TKA point to an increasing utilization of these technologies, custom cutting guides for example, saw a rise in use from 1.3 % of TKAs performed in 2009 to 6 % in 2012 [4].

While these technological aids promise to achieve improved component positioning, it is important to consider the clinical and functional implications, as well as the added costs and potential learning curve associated with adopting new technology. This review aims to highlight the current literature surrounding navigation and custom implants in total knee arthroplasty, in particular the effect on perioperative, radiographic, and clinical outcomes, to determine the role these new technologies may have in improving the patient experience with TKA.

## Computer navigation

In an effort to achieve a more accurate and reproducible method of implant positioning and with an overtone of improving clinical outcomes, computer-assisted arthroplasty techniques have been introduced. First described in the late 1990s, computer-assisted TKA has taken many forms, including both passive and active robotic-assisted systems, as well as patient-specific navigation instrumentation [10]. In a fundamental sense, robotic systems work by utilizing a digital image as a template and road map to guide the surgeon during the procedure. These navigation systems are classified into “open” and “closed” systems in which the navigation can be applied to any prosthesis or manufacturer in the former versus specific to only one prosthesis or manufacturer in the latter. Surgical instruments, such as cutting burrs and saws, can be incorporated into these systems, known as navigated freehand bone cutting, and matched to the image map to locate their exact position and provide haptic feedback during surgery. The construction of these image maps may take one of three forms, including preoperative imaging technique, intraoperative imaging technique, and image-free technique. In the preoperative technique, CT- or MRI-based images are obtained preoperatively to construct the surgical model. Intraoperative imaging systems use a modified fluoroscopy within the operating room suite at the time of surgery and can additionally provide real-time data to the system. An image-free technique uses surface probe akin to a coordinate measuring machine to detect the physical surface anatomy and to create an anatomical model through a surface registration process. This is currently the most commonly utilized technique for navigated TKA.

In addition to robotic- and radiographic-based navigation systems, gap balancing instruments and smart trial tibial liners have recently emerged into the marketplace. These devices contain microprocessor sensors which quantify relative load and contact position between the femur and tibia during the trial phase of the operation; the data is wirelessly transmitted to a graphic user interface for the surgeon to interpret. The intention of these devices is to eliminate the “feel” aspect of soft tissue gap balancing and provide a quantitative intraoperative measure of the load-bearing forces and component tracking dynamics. Clinical studies evaluating these devices are limited, and no long-term data is available assessing the impact these devices may have on clinical outcomes.

## Navigation outcomes

### Implant alignment

Assisting surgeons more consistently achieve prosthesis positioning, with reduction in outliers, is the most consistent reported advantage of computer navigation compared to conventional instrumentation. Meta-analysis by Mason et al. found that computer-assisted arthroplasty was superior to traditional instrumentation at achieving alignment within ±2° from the ideal in the mechanical axis, coronal plane, tibial slope, and sagittal femoral component positioning [9]. There was no difference for sagittal femoral alignment or tibial slope when the margin for error was ±3°. For mechanical alignment more specifically, this study found a malalignment of greater than 3° which occurred in only 9.0 % of patients in the CAS group versus 31.8 % of patients in the conventional TKA group. Bauwens et al., however, found no difference between navigated and instrumented TKA with regard to mean mechanical axis alignment but did corroborate the findings of fewer deviations from both greater than 3° and greater than 2° deviations [11]. A more recent meta-analysis by Hetaimish et al. of 23 randomized control trials again found that navigated TKA significantly improved prosthesis alignment [10]. Similar to the previous findings by Mason, Hetaimish additionally found that 30.1 % of patients in the conventional group had deviations in the coronal plane greater than 3° from neutral compared to 12.8 % in the navigation cohort [10]. While improvement in implant position has been shown with computer assistance, the results are heterogeneous and some controversy exists regarding the clinical application or significance of these findings.

### Perioperative metrics

Specific preoperative metrics which may have a role in postoperative outcomes include operative time, blood loss, and adverse events, and some trends have been borne out in the literature comparing traditional to computer assisted navigation.

Several studies have found a decrease in blood loss with CAS [12–15]. A randomized controlled trial by Kalairajah et al. reported less blood loss as measured by drain volume with navigated surgery [13]. Licini reported similar results looking specifically at femoral component navigation versus intramedullary femoral referencing, finding a decrease in hourly hemovac drain output, hemoglobin change, and estimated blood loss in the navigated group [15]. Other studies, however, have found no difference in total blood loss [16, 17].

Mean operative time and increased tourniquet time with computer-navigated knee arthroplasty have been well documented [11, 13, 14, 16, 18–21]. The meta-analysis by Bauwens, for example, noted a longer mean operative time by greater than 23 % for the navigated group, equating to an average 15 to 20 min longer procedure [11].

No significant difference has been shown with regard to postoperative hospital length of stay nor perioperative complications. Ajwani et al. showed no significant difference between navigation and conventional instrumentation in length of stay, and no difference in the rate of infection or deep vein thrombosis was found in systematic review by Zamora et al. [16, 22]. Intraoperative embolic load been shown to be higher in the traditionally instrumented group, associated with instrumentation of the femoral canal [22, 23]. Despite findings of greater pulmonary embolic loads, clinically significant embolic events are rare and found to likely be clinically insignificant.

### Clinical outcomes

Patient-related clinical outcome and satisfaction scores between computer-assisted and traditional arthroplasty techniques have not demonstrated consistently significant differences. Long-term survival of TKA after computer-assisted versus traditional instrumentation was characterized by de Steiger, in which for patients younger than 65, an overall lower rate of revision and lower revision rate for loosening or lysis was found in the navigation group [24]. The overall revision rate for all ages, however, was not significant between the two groups. Many studies find no difference in satisfaction, pain, or quality of life outcome; however, this conclusion is somewhat heterogeneously reported. No difference in Knee Society, WOMAC, SF-36 scores or patient satisfaction scores was found by Harvie et al. at 5-year follow-up, whereas Lehnen et al. reported higher patient satisfaction and improved WOMAC and Knee Society scores after navigated TKA [25, 26]. Significantly better Oxford Knee Scores were found by Blakeney et al. for navigated knee arthroplasty in which mechanical axis alignment was within 3° of neutral [27]. Cip et al. showed an improvement in Insall Knee Score and HSS Knee Score with computer-assisted technique but no improvement in WOMAC scores [28]. Hoffart et al. reported improved Knee Society Scores for navigated TKAs, where as Kim et al. could not find differences in Knee Society Scores [19, 29]. Based on the results of 5 studies, the clinical practice guidelines put forth by the American Academy of Orthopaedic Surgeons recommend against navigation on the basis that at follow-up greater than 90 days, there were no differences in patient-reported quality of life outcomes defined by the EQ-5D and SF-36 Mental Component Summary, patient-reported knee function defined by the Oxford Knee Score, Knee Society Score, and WOMAC, and pain defined by the WOMAC score [30].

An interesting, and possibly not well-quantified, use of navigation is in the application of navigation to minimally invasive total knee arthroplasty. The argument behind minimally invasive approaches include decreased blood loss, less pain, faster return to function, and a shorter hospital stay, all ultimately improving clinical and satisfaction outcomes. Khakha et al. demonstrated a shorter length of stay and improvement in knee society scores at up to 2 years of follow-up, with no compromise in implant positioning comparing traditional computer-assisted arthroplasty to minimally invasive computer-assisted arthroplasty, defined by an incision less than 12 cm through a mini-midvastus approach [31]. Often the challenge with the application of a minimally invasive philosophy to TKA is the decrease in prosthesis alignment accuracy, subject to the compromised exposure afforded by the minimally invasive approach. The argument to using navigation is that it does not force the surgeon to rely on a wide exposure for identification of anatomic landmarks and therefore can take advantage of both the accuracy of navigation with minimal surgical dissection.

## Patient-specific instrumentation

While the aim of patient-specific instrumentation (PSI) is that of computer navigation—improving the alignment and thus the outcome of TKA—PSI seeks to accomplish its goal in the preoperative period instead of in the operating suite. Currently, seven orthopaedic implant manufacturers offer PSI systems (Biomet, ConforMIS, DePuy, Medacta, Smith & Nephew, Wright Medical Technology, and Zimmer). Preoperative three-dimensional imaging, either CT scan or MRI depending on the manufacturer, is used to model a patient’s anatomy and design an individual surgical plan with respect to positioning, alignment, and resection. Once a surgeon approves the plan, cutting blocks or pin guides, depending on manufacturer, are rendered and shipped to the hospital, usually in sterile packaging acceptable for the operating room. Pin guides sit on the anterior distal femur and proximal tibia to set the placement of pins into the femur and tibia. Onto these pins, manufacturer-provided cutting-jigs are placed. Whereas custom cutting guides are pinned directly into place and contain cutting slots through which a standard saw is used. It is important to note that PSI systems will not perform gap balancing or soft tissue releases, which are crucial to the success of the surgery. Likewise, tibial component rotation and implant fixation, as well as patellar preparation, remain the responsibility of the surgeon.

### Perioperative metrics

In theory, PSI should decrease surgical time by eliminating intraoperative decision-making, since component sizing, rotation, and femoral and tibial resection are predetermined. Despite this theoretical efficiency, the results of whether PSI does decrease time spent in the OR compared to conventional TKA have been disparate. Boonen et al. noted a 10-min reduction in operative time for PSI TKA compared to conventional intramedullary TKA [32]. DeHaan et al., in a series of 60 knees, found 20.4-min reduction in surgical time in the PSI cohort [33]. Renson et al. reported an 8.6-min reduction in PSI knees [34]. Noble et al. reported a mean surgical duration 6.7 min shorter with PSI than conventional instrumentation, 121.4 and 128.1 min respectively [35]. Vide et al. randomly assigned 95 patients to either TKA with PSI or conventional methods and reported an 18-min reduction in operative time [36]. A 5-min reduction in operative time was reported by Chareancholvanich et al. in a randomized control trial of 80 patients, a finding deemed clinically irrelevant by the authors [37]. Conversely, Hamilton et al. found no difference in surgical time between 56 knees randomly assigned to either conventional TKA or PSI [38]. Stronach et al. found mean surgical times of 59.1 and 59.2 min for conventional TKA and PSI, respectively [39]. A recent meta-analysis by Voleti et al. looking at nine studies and 957 patients found a trend toward decreasing operative times with a mean of 5 min per patient; however, this was not statistically significant [40].

### Implant alignment

As stated previously, achieving accurate and specific alignment is one of the primary goals of TKA, as such, a large amount of PSI published literature concerns alignment accuracy. Ng et al. retrospectively reviewed 569 TKAs performed with PSI and 155 with manual instrumentation using postoperative long-leg radiographs and found significantly less hip-knee-ankle angle outliers (±3°) with PSI than with manual instrumentation, 9 to 22 % respectively [41]. Heyse et al. compared 46 MRI-based PSI TKAs and 48 conventional TKAs reporting more outliers (>3°) in terms of femoral rotation for conventional TKAs [42]. In a randomized control trial, Noble et al. noted a mechanical axis significantly closer to zero in the PSI group, 1.7° versus 2.8° [35]. Chareancholvanich et al. found no difference in tibiofemoral or femoral component alignment but did find a difference in tibial component alignment with PSI being closer to neutral than standard instrumentation [37]. Conversely, Hamilton et al. reported no difference in mechanical axis or component alignment for 56 randomized TKAs [38]. Two recent meta-analyses investigated the accuracy of alignment. Jiang et al. compiled 18 studies with 2417 patients, demonstrating no significant difference in the number of outliers in mechanical axis as well as coronal, sagittal, and axial alignment [43]. Mannan et al., however, did note favorable femoral rotational outcomes in a meta-analysis of 6 studies on a total of 444 knees [44].

### Computed tomography versus magnetic resonance templating

Both CT and MRI can be used for preoperative templating; however, recent studies suggest that MRI-based may be superior. Asada et al. divided 40 patients equally between CT- and MRI-guided PSI. They noted significantly shorter OR times with MRI than CT, 109.2 min compared to 129.5 min, but no difference in axial, coronal, or sagittal alignment [45]. Frye et al. evaluated 23 CT-based TKAs and 27 MRI-based TKAs, finding significantly more mechanical axis outliers in CT-based TKAs than in MRI-based TKAs [46].

### Plan accuracy

For PSI to be efficient, the preoperative plan and custom guides must be precise; if not, conventional TKA steps cannot be skipped, and secondary checks of resection depth, component size, and rotation must be performed, adding time to the procedure. Multiple studies have shown that this may be the case with custom instrumentation. In a series of 66 knees, Stronach et al. reported a total of 161 intraoperative changes, 2.4 changes/knee, of which the majority were improvements to the preoperative surgical plan [47]. Scholes et al. found an error between intraoperative measurements and the preoperative plan in 27 % of 30 knees [48].

### Cost

Although the literature regarding PSI and surgical efficiency has been equivocal, multiple studies have shown that PSI does reliably decrease the number of trays needed for TKA. Hamilton et al. reported a mean of 2.5 trays required for PSI compared to 7.3 trays for conventional TKA [38]. Noble et al. found a similar reduction in the number of trays in PSI compared to conventional TKA, with means of 4.5 and 7.3 respectively [35]. Renson et al. found a 50 % reduction in instrumentation trays per case in a series of 131 TKAs, of which 71 were PSI [34].

Despite the reduction in the number of trays and potential for decreased surgical time, the costs of preoperative imaging and the manufacturing costs can offset any savings. Barrack et al. evaluated 200 consecutive TKAs, of which 100 were PSI, and found that while both operative time and the number of trays were reduced, the mean savings per patient was $322 which did not offset the mean cost of the preoperative MRI and cutting blocks, approximately $1500 [49]. Thienpont et al. evaluated 80 TKAs and noted the indirect costs, i.e., manufacturing and imaging, of PSI average 40 % of the total cost [50].

### Clinical outcomes

Ultimately, the fundamental question for whether to adopt PSI is not whether it saves time, money, or improves alignment but whether it benefits the patient and improves outcomes. Abane et al., in a multicenter randomized trial of 126 patients, found no difference between conventional TKA and PSI in postoperative alignment, blood loss, length of stay, Oxford Knee Scores, and Knee Society Scores [51]. Vundelinckx et al. found similar results in 31 patients with PSI knees. When compared to a conventional control group, no statistically significant difference could be found in postoperative pain, satisfaction, functional outcome, hospital stay, blood loss, radiographic alignment, or precision of bone cuts [52]. Additionally, Abdel et al. performed a 3-dimensional gait analysis on 40 patients randomized to conventional TKA or PSI and reported no difference in functional or gait parameters at 3 months [53]. Chen et al. compared 28 PSI to 29 conventional TKAs and noted no difference in the Knee Society Function Score, Oxford Knee Score, and SF-36 at 2-year follow-up. While the study did find a significant difference in Knee Society Knee Score in the PSI group at 2 years, the improvement was comparable between the two groups [54]. To our knowledge, there are no studies investigating the survivorship of PSI TKA compared to that of conventional TKA.

## Discussion

Advances in computer and manufacturing technology have revolutionized the way in which we conduct our daily lives and practice of medicine; this is no more true than in the field of orthopaedics, rooted in a history of applying and evolving new technologies to provide more consistent and reproducible quality-of-life-altering treatments. Despite significant advances and development in computer- and robotic-assisted technology, however, the reality is that while computer-assisted surgical systems seem to allow for more consistent alignment with fewer outliers, significant changes in patient outcomes have not been actualized. Similarly, despite the potential for improved accuracy and efficiency, the majority of published literature does not support the use of PSI over conventional TKA in improving outcomes.

It needs to be acknowledged, however, that the majority of literature concerning both PSI and computer-assisted arthroplasty are done in an academic setting, with fellowship-trained joint surgeons. For these individuals, the equivocal results regarding PSI or navigation compared to conventional TKA could be a product of their expertise and familiarity with knee arthroplasty. In the hands of a community surgeon who is not quite as facile, there may be a benefit for these technologies that has not yet been well established in the literature.

## Conclusions

While the clinical benefits of computer-aided surgery or PSI may not be actualized as it was intended, surgeons should not be wary of new technologies. It is with the adoption and subsequent critical evaluation of technology that innovation can occur. Despite equivocal success in the average patient, both navigation and PSI have shown to be of benefit in extra-articular deformity cases, or in cases in which conventional techniques cannot be applied, such as a patient with intramedullary implants already in place. Thienpont et al. reported restored limb alignment and improved functional scores in patients without access to the intramedullary canal who underwent TKA with PSI (Knee, 2013) [55]. Several others have similarly reported success using navigation in cases where deformity or prior hardware limited traditional instrumentation [56–58].

Extra-clinical benefits, moreover, may not have yet been well defined for these technologies. The preoperative imaging of PSI, for example, can provide a wealth of anthropometric data for research purposes, in particular the rotational alignment of the distal femur [59]. Navigation similarly opens the door to an array of research, teaching, and surgical documentation opportunities.

While navigation and customized implants have found recent interest in the knee arthroplasty marketplace, in a broad sense and in their current forms, these technologies have yet to reach their full potential in improving outcomes and patient experience.
