# Erratum to: Improving outcomes in total knee arthroplasty—do navigation or customized implants have a role?

**DOI:** 10.1186/s13018-016-0453-3

**Published:** 2016-10-14

**Authors:** Matthew D. Beal, Dimitri Delagrammaticas, David Fitz

**Affiliations:** Northwestern University Feinberg School of Medicine, Chicago, USA

After publication of the original article [1], it was noted that one of the author’s names was presented incorrectly. The correct name of the author (Dimitri Delagrammaticas) has been updated in the original article, and published in this Erratum for quick reference. We apologise for any confusion this may have caused.
